# Grandparental immune priming in the pipefish *Syngnathus typhle*

**DOI:** 10.1186/s12862-017-0885-3

**Published:** 2017-02-07

**Authors:** Anne Beemelmanns, Olivia Roth

**Affiliations:** Evolutionary Ecology of Marine Fishes, Helmholtz-Centre for Ocean Research Kiel (GEOMAR), Düsternbrooker Weg 20, 24105 Kiel, Germany

**Keywords:** Grandparental effects, Immune priming, Epigenetic inheritance, Immune defense, Host-parasite interaction, Gene expression

## Abstract

**Background:**

Phenotypic changes in response to environmental influences can persist from one generation into the next. In many systems parental parasite experience influences offspring immune responses, known as transgenerational immune priming (TGIP). TGIP in vertebrates is mainly maternal and short-term, supporting the adaptive immune system of the offspring during its maturation. However, if fathers and offspring have a close physical connection, evolution of additional paternal immune priming can be adaptive. Biparental TGIP may result in maximized immunological protection. Here, we investigate multigenerational biparental TGIP in the sex-role reversed pipefish *Syngnathus typhle* by exposing grandparents to an immune challenge with heat-killed bacteria and assessing gene expression (44 target genes) of the F2-generation.

**Results:**

Grandparental immune challenge induced gene expression of immune genes in one-week-old grandoffspring. Similarly, genes mediating epigenetic regulation including DNA-methylation and histone modifications were involved in grandparental immune priming. While grand-maternal impact was strong on genes of the complement component system, grand-paternal exposure changed expression patterns of genes mediating innate immune defense.

**Conclusion:**

In a system with male pregnancy, grandparents influenced the immune system of their grandoffspring in a sex-specific manner, demonstrating multigenerational biparental TGIP. The involvement of epigenetic effects suggests that TGIP via the paternal line may not be limited to the pipefish system that displays male pregnancy. While the benefits and costs of grandparental TGIP depend on the temporal heterogeneity of environmental conditions, multigenerational TGIP may affect host-parasite coevolution by dampening the amplitude of Red Queen Dynamics.

**Electronic supplementary material:**

The online version of this article (doi:10.1186/s12862-017-0885-3) contains supplementary material, which is available to authorized users.

## Background

In sexually produced offspring, genotypes are determined by both maternal and paternal genetic contributions. An offspring phenotype is also influenced by a plethora of environmental factors experienced during its ontogeny and by its parents [1–3]. Such transgenerational plasticity of phenotypes is often adaptive, can promote efficient and rapid acclimatization to environmental changes, and even has the potential to modify evolutionary dynamics [4–6]. Anti-predator defenses [7], tolerance of abiotic environmental change [8–10], and induced disease resistance in offspring [11, 12] are amongst the most studied transgenerational effects that are not inherited via DNA, but through a diversity of alternative mechanisms [13].

The transmission of parental parasite experience that subsequently leads to an enhanced offspring immune defense is known as transgenerational immune priming (TGIP) [14–19]. TGIP enables a faster or stronger offspring immune reaction that matches the current parasite environment [20]. Environmental variation can result in heterogeneous parasite distributions across environments [21] persisting through host generations. Under such matching environmental conditions where host-dispersal is limited and hosts have a long lifespan, selection for TGIP is predicted to be strong and evolutionarily adaptive [22, 23].

In vertebrates, studies of TGIP have mainly focused on the transfer of maternal antibodies [14, 17, 24], while also substances of the innate immune system are involved [25–27]. Usually mothers deposit immune defense components into the eggs, transfer them during development (e.g. via the placenta), or, in mammals, after birth via lactation [14, 17, 28]. The classical view is that male sperm only contributes to heredity, i.e. via DNA, to the offspring. Considering recently discovered hereditary mechanisms that are not based on the pure DNA sequence itself, like DNA-methylation, histone acetylation pattern or tRNA, this view is challenged into question [29–32]. Recently, a growing number of examples indicate influences beyond pure transfer of DNA via sperm on offspring and, thus, underline the fathers’ role beyond the determination of the offspring genotype [32–38]. In an invertebrate system with only an ejaculate-based connection between father and offspring, the paternal environment influences offspring immune phenotype [36, 39]. Further, a strong paternal contribution to immune phenotype of progeny was found in vertebrates with intense paternal care or investment [19, 35, 40, 41].

The advantages of TGIP in vertebrates were considered to be strongest during early life stages by strengthening the developing adaptive offspring immune system, with the effect fading upon maturation [42, 43]. However, some ecological conditions may select for multigenerational TGIP modulating immune responses beyond the F1-generation [44]. Persistent TGIP should be favoured when the parasite environment is stable over time and, hence, across host generations. So far, our knowledge about TGIP in vertebrates past the early phase of an offspring’s life is limited. Only scarce evidence supports the existence of TGIP beyond the maturation of the adaptive immune system of vertebrates [45, 46], while in invertebrates TGIP can apparently cross the borders of more than just one generation [47].

To explore the potential for long lasting and multigenerational effects on immunity, we experimentally assessed grandparental TGIP in the sex-role reversed pipefish *Syngnathus typhle.* In this fish species, males have evolved a unique placenta-like structure [48]. Not mothers but fathers are thus the pregnant sex [49]. During male pregnancy, embryos are provided with nutrients and oxygen over this placenta-like structure [50–53], which may mechanistically enable a paternal investment into offspring immune defense. Usually in teleosts, females transfer immune components such as immunoglobulins, complement components, lectins, lysozymes and soluble antimicrobial peptides across follicle cells during the early stage of vitellogenesis into the oocyte [26, 54]. However, apart from maternally derived immunity syngnathids profoundly rely on supplemental paternal immune priming [19, 41]. As such, the pipefish system was chosen as here not only mothers but also fathers can induce offspring immune response over biparental TGIP [19, 41]. So far, it was considered that TGIP in vertebrates is only of major importance during early development, to bridge the time of maturation of the acquired immune system, when selection pressure due to high mortality is greatest [55]. In contrast, recent studies indicate that in *Syngnathus typhle* the persistence of immune priming lasts past the maturation of the adaptive immune system in four-month-old juveniles [41], coupled with a high degree of bacteria specificity [40]. In the current study we aimed to address the impact of biparental parental immune priming beyond the generation border, affecting the immune dynamics of the grandoffspring generation.

**Fig. 1:**
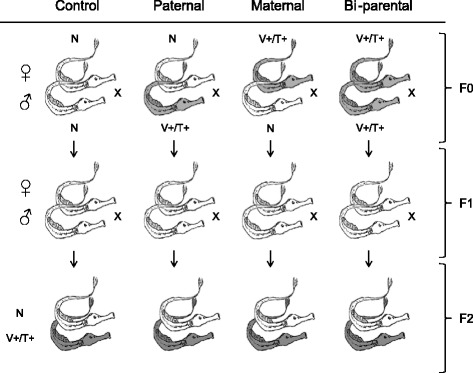
Experimental design. The grandparental generation (F0) was vaccinated using a combination of heat-killed immunological novel *Vibrio* spp. and *Tenacibaculum maritimum* (F0-bacteria), or were left naïve (F0-N) as control. Immune-challenged mature pipefish were used in following mating design: 1. Control: [♀F0-naïve x ♂F0-naïve﻿]; 2. Paternal: [♀F0-﻿naïve x ♂F0-bacteria]; 3. Maternal: [♀F0-bacteria x ♂F0﻿-naïve] and 4. Biparental: [♀F0-bacteria x ♂F0-bacteria] and kept according to their mating pairs (families) in separate 36 × 80 L semi-flow through aquaria (16 family replicates per parental bacteria treatment and eight per control group; 56 families). F1-individuals were crossed within former parental treatment groups but left immunologically naïve (out of each of the four grandparental treatment groups five families were chosen to do F1-crosses resulting in 20 F1-families). In spring 2014, F2-juveniles were exposed one-week post birth to the same heat-killed *Vibrio* (F2-V+) and *Tenacibaculum* (F2-T+) bacteria used for the F0-generation or left naïve (F2-N) (per F1-crossing four families produced F2-offspring resulting in 16 F1-families). Out of each family 12 individuals were chosen for the direct immune challenge. Per F2-offspring treatment (F2-V+, F2-T+, F2-N) four individual replicates were used; resulting in a total of 192 samples

Mature pipefish males and females (F0-generation) were exposed to two heat-killed bacteria (*Vibrio* spp. and *Tenacibaculum maritimum*) or a control prior to mating in a fully reciprocal mating design (Fig. 1). By leaving the F1-generation untreated, we were able to examine grandparental immune priming effects in the F2-offspring while challenging them with the same bacteria treatment as their grandparents (F0-generation). For the evaluation of grandparental sex-specific influences on grandoffspring immunity, either only grandmothers, only grandfathers or both grandparents (grand-biparental) were expsed to the bacteria treatment. In the F2-generation we assessed expression of 44 target genes functionally associated to different pathways of the immune system (innate and adaptive immune system, complement component system) and epigenetic regulation processes (DNA-methylation and histone modifications), to test whether complementing sex-specific contribution as previously found to exist for the F1-generation [40] may have been trans generationally maintained over two generations. Here, we found strong grandparental effects that influenced the immune gene expression of grandoffspring upon bacterial exposure. This grandparental TGIP is sex-specific (grandfather vs. grandmother) with regard to immune pathway activation and the involvement of epigenetic regulation genes.

## Results

By using multivariate data analyses differential gene expression patterns were explored in one-week-old F2-juveniles upon the applied F0-grandparental bacteria treatment (Vibrio: ‘V+’ and Tenacibaculum: ‘T+’ bacteria) in contrast to the naïve control group (Naïve: ‘N’). We evaluated with a Permutational Multivariate Analysis of Variance (PERMANOVA) whether gene expression (44 target genes) of F2-juvenile pipefish revealed grandparental sex-specific influences (‘F0-sex’) and grandoffspring bacteria treatment effects (‘F2-bacteria’) including their interaction (‘F0-sex x F2-bacteria’), while setting the family structure as random term. The multivariate PERMANOVA model was based on an Euclidean distance matrix and applied for 29 immune genes and 15 genes associated to epigenetic regulation, but also divided into following specific functional gene categories: (i) innate immune system (13 genes connected to the pro-inflammatory immune response), (ii) adaptive immune system (eight genes associated to the antibody-mediated immune defense), (iii) innate and adaptive immune genes (five genes connected to both immune pathways), (iv) complement system (three complement component genes that assist the antibody and phagocytic cell mediated immune response), (v) DNA methylation (five genes coding for DNA-methyltransferases), (vi) histone de/methylation (four histone de/methyltransferase genes), (vii) histone deacetylation (three histone deacetylation genes), and (vii) histone acetylation (two histone acetylation genes) [41]. Finally, we evaluated the contribution of variance explained by each target gene to identify central genes driving the grandparental bacteria treatment effect.

### Differences between grand-paternal and/or grand-maternal immune priming (F0-sex bacteria treatment effect and F0-sex x F2-bacteria interaction)

#### Immune gene expression (29 genes-total)

**Fig. 2:**
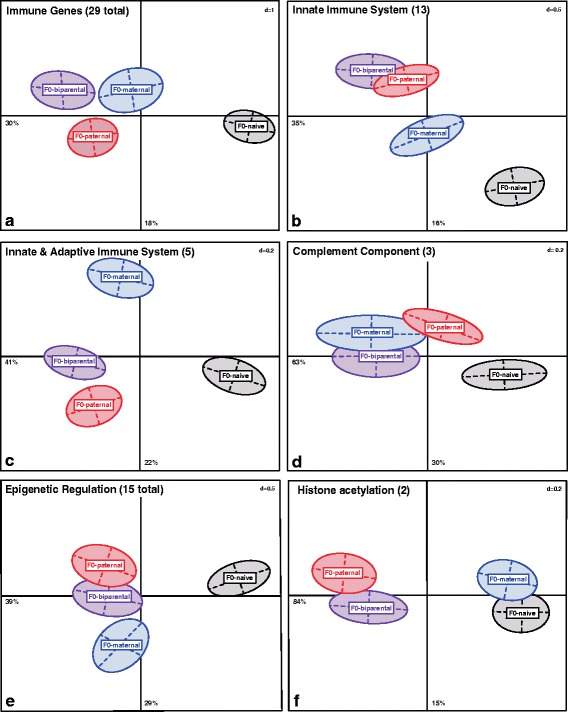
Principle Component Analysis (PCA) depicting the grandparental bacteria treatment effect on gene expression of one-week-old F2-juveniles. PCA to visualize gene categories revealing a significant different gene expression profiles per grandparental control (F0-control), grand-paternal (F0-paternal), grand-maternal (F0-maternal) and grand-biparental (F0-biparental) bacteria treatment groups (Panels﻿﻿ a-f) on relative gene expression data (−∆Ct-values) using an Euclidean distance matrix (*N* = 192). Panel** a** all immune genes (29 genes-total), Panel** b** genes of the innate immune system (13 ﻿genes), Panel **c** genes of the innate & adaptive immune system (5 genes); Panel** d** complement component genes (3 ﻿genes); Panel** e** epigenetic regulation genes (15 genes-total) and Panel** f** histone acetylation genes (2 ﻿genes). The variance in percentage (%) explained by the respective principle coordinates (PCs) is indicated below (for PC1) and besides (for PC2) the corresponding axis. The size (cm) of the grid is indicated by `d´ for dimension in the upper right corner

Based on 29 immune genes, we found marked and significantly different expression profiles among all four grandparental sex-specific bacteria treatment groups (PERMANOVA-*immune*: F_3,174_ = 6.82, *p* < 0.001, Table 1). We applied a PCA and ANOSIM analysis focusing on grandparental sex specific immune priming effects (F0-sex) (Fig. 2a, Table 2). Along the Principle Component (PC) one, the grandparental control group (F0-N) clusters opposed to all other three grandparental treatments, demonstrating a strong grand-paternal (F0-Pat), grand-maternal (F0-Mat) and grand-biparental (F0-Bi) treatment effect on F2-offspring immune gene expression (Fig. 2a). All four grandparental treatment groups were significantly different from each other (ANOSIM-*immune*: F0-Bi vs. F0-Mat *p* = 0.004; F0-Bi vs. F0-Pat *p* = 0.003; F0-Mat vs. F0-Pat *p* = 0.007; F0-Bi vs. F0-N *p* = 0.001; F0-Mat vs. F0-N *p* = 0.001; F0-Pat vs. F0-N *p* = 0.001, Table 2). As the grand-paternal and grand-maternal treatment groups are clustering on the same level in the PCA without overlapping centers of gravity whereas the grand-biparental treatment group clusters further apart, this pattern indicates similar grand-maternal and grand-paternal influences on immune gene expression of F2-juveniles (Fig. 2a). In addition, post hoc pairwise comparisons of the significant F0-sex x F2-bacteria interaction (PERMANOVA-*immune*: F_6,174_ = 1.32, *p* = 0.009, Table 1) demonstrate grandparental sex-specific influences between grand-paternal (F0-Pat) and grand-maternal (F0-Mat) bacteria exposure. Although the F2-generation received a bacterial immune treatment (F2-T+, F2-V+) grandparental sex-specific influences were dominating (ANOSIM-*immune*: F0-Mat x F2-bacteria (V+ or T+) vs. F0-Pat x F2 bacteria (V+ or T+) *p* < 0.030, Table 2). The combination of grand-maternal and grand-paternal exposure in a grand-biparental treatment did not differ from the single grandparental effects, designating an intermediate impact of grandmothers and grandfathers (ANOSIM-*immune*: F0-Bi x F2-bacteria (V+ or T+) vs. F0-Pat x F2 bacteria (V+ or T+) *p* > 0.050; F0-Bi x F2-bacteria (V+ or T+) vs. F0-Mat x F2 bacteria (V+ or T+) *p* > 0.050, Table 2).

**Table 1:** Results from 2-way PERMANOVA analysis of gene expression ﻿of one-week-old F2-juveniles

Gene categories	Model	F0-sex			F2-bacteria			F0-sex x F2-bacteria			Size	
	R^2^	F.Model	Pr(>F)		F.Model	Pr(>F)		F.Model	Pr(>F)		F.Model	Pr(>F)
Immune genes [29 genes-total]	0.83	6.82	**> 0.001**	***	3.08	**>0.001**	***	1.32	**0.009**	**	1.13	0.641
Innate immune genes [13 genes]	0.83	6.67	**0.004**	**	2.01	**0.026**	*	1.87	**0.007**	**	1.26	0.431
Adaptive immune genes [8 genes]	0.80	1.53	0.108		1.00	0.184		0.99	0.100		1.71	0.521
Innate & Adaptive genes [5 genes]	0.84	5.88	**0.001**	**	5.47	**>0.001**	***	0.71	0.622		0.86	0.460
Complement component genes [3 genes]	0.86	5.31	**0.017**	*	3.66	**0.001**	***	0.80	0.237		0.71	0.790
Epigenetic genes [15 genes-total]	0.85	6.63	**0.035**	*	1.64	**0.030**	*	1.22	**0.029**	*	0.62	0.894
DNA-methylation genes [5 ﻿genes]	0.85	6.09	0.061	.	2.26	**0.022**	*	1.18	0.081	.	0.84	0.812
Histone de/methylation genes [4 ﻿genes]	0.89	4.16	0.195		0.68	0.516		1.33	0.082	.	0.20	0.844
Histone deacetylation genes [3 ﻿genes]	0.86	5.65	0.079	.	1.23	0.126		1.21	0.060	.	1.39	0.621
Histone acetylation genes [2 ﻿genes]	0.78	12.47	**0.035**	*	2.03	**0.019**	*	1.09	0.065	.	0.15	0.896
Degrees of Freedom		DF = 3			DF = 2			DF = 6			DF = 1	
Residual Degrees of Freedom	174											
Total Degrees of Freedom	186	*For further details see Additional file* 1 *: Table S1*										

Multivariate PERMANOVA analysis to assess the effect and interaction of the two fixed factors F0-sex and F2-offspring, size as covariate and family as strata term on relative gene expression values (−∆Ct-values). Each analysis was based on an Euclidean distance matrix with *p*-values obtained by 10000 permutations. Significant *p*-values are marked in bold letters and asterix symbol (significance code: <0.001***, 0.001**, 0.01*, 0.1 > *p*-value ≥ 0.05 trend ●). R^2^ value indicate the percentage of variance explained by the model

**Table 2:** Results from PERMANOVA and ANOSIM analysis of one-week-old F2-juveniles per functional gene categories

F2-juveniles (One-week-old)	Immune genes [29 total]	Innate genes [13]	Adaptive genes [8]	Innate & Adaptive genes [5]	Complement component genes [3]	Epigenetic genes [15 total]	DNA-methylation genes [5]	Histone-de/methylation genes [4]	Histone deacetlyation genes [3]	Histone acetylation genes [2]
F0-sex (DF = 3)	**<0.001**	**0.004**	*ns*	**0.001**	**0.017**	**0.035**	*ns*	*ns*	*ns*	**0.035**
ANOSIM-Global R	0.115	0.12	*ns*	0.104	0.054	0.088	*ns*	*ns*	*ns*	0.088
Significance level	0.1%	0.1%	*ns*	0.1%	0.1%	0.1%	*ns*	*ns*	*ns*	0.1%
F0-Bi, F0-Mat	**0.004**	**0.001**	*ns*	**0.003**	*ns*	**0.027**	*ns*	*ns*	*ns*	**0.008**
F0-Bi, F0-Pat	**0.003**	*ns*	*ns*	**0.002**	**0.009**	*ns*	*ns*	*ns*	*ns*	*ns*
F0-Mat, F0-Pat	**0.007**	**0.001**	*ns*	**0.001**	*ns*	**0.001**	*ns*	*ns*	*ns*	**0.001**
F0-Bi, F0-N	**0.001**	**0.001**	*ns*	**0.001**	**0.002**	**0.001**	*ns*	*ns*	*ns*	**0.009**
F0-Mat, F0-N	**0.001**	**0.001**	*ns*	**0.002**	**0.002**	**0.009**	*ns*	*ns*	*ns*	*ns*
F0-Pat, F0-N	**0.001**	**0.001**	*ns*	**0.001**	*ns*	**0.001**	*ns*	*ns*	*ns*	**0.007**
F2-bacteria (DF = 2)	**<0.001**	**0.026**	*ns*	**<0.001**	**0.001**	**0.030**	**0.022**	*ns*	*ns*	**0.019**
ANOSIM-Global R	0.022	0.018	*ns*	0.026	0.024	0.004	0.011	*ns*	*ns*	0.009
Significance level	7.4%	11.5%	*ns*	2.1%	5.9%	31.9%	20.9%	*ns*	*ns*	72.9%
F2-V+, F2-T+	*ns*	*ns*	*ns*	*ns*	*ns*	*ns*	*ns*	*ns*	*ns*	*ns*
F2-V+, F2-N	**0.022**	**0.011**	*ns*	**0.006**	**0.024**	**0.005**	**0.005**	*ns*	*ns*	*ns*
F2-T+, F2-N	**0.021**	**0.016**	*ns*	**0.030**	*ns*	**0.049**	**0.049**	*ns*	*ns*	**0.05**
F0-s﻿ex x F2-b﻿acteria (DF = 6)	**0.009**	**0.007**	*ns*	*ns*	*ns*	**0.029**	*ns*	*ns*	*ns*	*ns*
ANOSIM-Global R	0.105	0.103	*ns*	*ns*	*ns*	0.074	*ns*	*ns*	*ns*	*ns*
Significance level	0.1%	0.1%	*ns*	*ns*	*ns*	0.1%	*ns*	*ns*	*ns*	*ns*
F0-Mat/F2-V+, F0-Pat/F2-V+	**0.036**	**0.030**	*ns*	*ns*	*ns*	**0.004**	*ns*	*ns*	*ns*	*ns*
F0-Mat/F2-V+, F0-Pat/F2-T+	**0.049**	**0.007**	*ns*	*ns*	*ns*	**0.004**	*ns*	*ns*	*ns*	*ns*
F0-Mat/F2-T+, F0-Pat/F2-V+	**0.025**	**0.028**	*ns*	*ns*	*ns*	**0.004**	*ns*	*ns*	*ns*	*ns*
F0-Mat/F2-T+, F0-Pat/F2-T+	**0.033**	**0.04**	*ns*	*ns*	*ns*	**0.010**	*ns*	*ns*	*ns*	*ns*
*For further details see ﻿Additional file* 1 *: Table S2*										

Multivariate ANOSIM was performed following significant PERMANOVA effects to assess differences in the gene expression profiles per treatment groups applying pairwise comparison on relative gene expression data (−∆Ct-values) based on a Euclidean distance matrix and 10000 permutations. Pairwise comparison was conducted for following two fixed factors and their interactions: ‘F0-sex’ (grandparental (F0-Bi), grand-maternal (F0-Mat), grand-paternal (F0-Pat), grandparental control (F0-N)) and ‘F2-bacteria’ (F2-offspring control (F2-N), F2-offspring *Vibrio* (F2-V+) and *Tenacibaculum* (F2-T+) bacteria treatment)﻿

#### Innate immune genes (13 genes)

F2-offspring innate immune gene expression profile differentiated depending on whether grandmothers, grandfathers, both or none were immune-challenged (PERMANOVA-*innate*, F_3,174_ = 6.67, *p* = 0.004, Table 1, Fig. 2b). Likewise a significant F0-sex x F2-bacteria interaction (PERMANOVA-*innate*: F_6,174_ = 1.87, *p* = 0.007, Table 1) proposes grandparental sex-specific influences, outweighing the F2-bacteria treatment (ANOSIM-*innate*: F0-Mat x F2-bacteria (V+ or T+) vs. F0-Pat x F2-bacteria (V+ or T+) *p* < 0.040, Table 2). In contrast to previous findings of combined immune genes, F2-offspring of the grand-paternal bacteria treatment display exactly the same expression profile as F2-offspring from the grand-biparental bacteria treatment (ANOSIM-*innate*: F0-Pat vs. F0-Bi *p* = 0.096, Table 2 & Additional file 1: Table S2, Fig. 2b). In the PCA grand-paternal and grand-biparental groups have remarkable overlapping centers of gravity indicating, that the grand-biparental group is more similar to the grand-paternal group than to the grand-maternal group which clusters further apart (ANOSIM-*innate*: F0-Bi vs. F0-Mat *p* = 0.001, F0-Bi vs. F0-Pat *p* = 0.096, F0-Mat vs. F0-Pat *p* = 0.001, Table 2﻿,﻿ Additional file 1: Table S2, Fig. 2b). Nevertheless, the grand-maternal treatment group is set apart from the F0-naïve control treatment (ANOSIM-*innate*: F0-Mat vs. F0-N *p* = 0.001, Table 2, Fig. 2﻿b), implying that the grand-maternal bacterial exposure still reveals a diminished effect. These findings denote that the bacterial environment experienced by the grandfathers drives the grand-biparental impact on genes of the innate immune system to a larger extent.

**Fig. 3:**
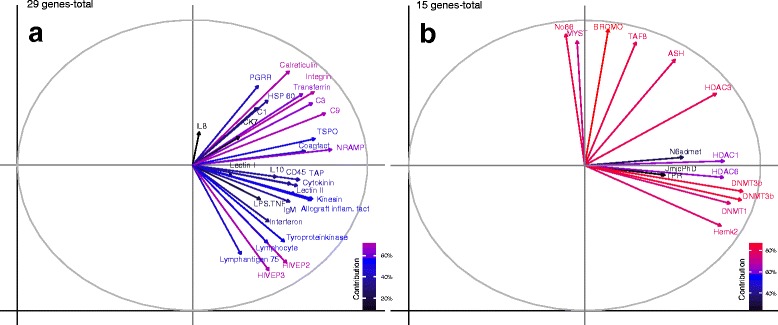
Factor maps to demonstrate the contribution of variance retained by each principal component for immune genes (29 genes-total) and epigenetic regulation genes (15 genes-total) of one-week-old F2-juveniles. The response variables (genes) are symbolized by arrows whereby the length of the arrow is directional proportional with the contribution of variance of each gene to the total variability. The colour gradient in the left corner highlights the most important genes in explaining the variation (contribution %) retained by the principle components calculated according to [97]

#### Adaptive immune genes (eight genes)

Offspring adaptive immune gene expression did not reveal significant grandparental sex-specific effects (PERMANOVA-*adaptive*, F_3,174_ = 1.53, *p* = 0.108, Table 1) nor F0-sex x F2-bacteria interaction effects (PERMANOVA-*adaptive*, F_6,174_ = 0.99, *p* = 0.100, Table 1).

#### Innate & Adaptive immune genes (five genes)

Five genes associated to both innate and adaptive immune response showed grandparental sex-specific treatment effects (PERMANOVA-*innate&adaptive:* F_3,174_ = 5.88, *p* = 0.001, Table 1, Fig. 2c). F2-juveniles of grand-maternal and grand-paternal treatment groups revealed a different expression profile from one another (ANOSIM-*innate&adaptive:* F0-Mat vs. F0-Pat *p* = 0.001, Table 2), but also from the grand-biparental treatment (ANOSIM- *innate&adaptive*: F0-Bi vs. F0-Pat *p* = 0.002; F0-Bi vs. F0-Mat *p* = 0.003, Table 2) and the control group (ANOSIM-*innate&adaptive:* F0-Mat vs. F0-N *p* = 0.002; F0-Pat vs. F0-N *p* = 0.001, Table 2 & Additional file 1: Table S2, Fig. 2c).

#### Complement component system (three genes)

The expression of complement component genes (*C3, C9* and *C1Q-sco*) that mediate between innate and adaptive immune system and also complement the antibody-mediated immune response, showed grandparental sex-specific influences (PERMANOVA-*complement:* F_3,174_ = 5.31, *p* = 0.017, Table 1, Fig. 2d). The complement component factors were impacted predominantly by the grand-maternal treatment (ANOSIM-*complement:* F0-Mat vs. F0-N *p* = 0.002, Table 2) but not by the grand-paternal treatment (ANOSIM-*complement:* F0-Pat vs. F0-N *p* = 0.124, Additional file 1: Table S2). As the grand-maternal and grand-biparental groups are not significantly different from each other and have the same center of gravity in the PCA (ANOSIM-*complement*: F0-Bi vs. F0-Mat = 0.168, Table 2 & Additional file 1: Table S2; Fig. 2d) the gene expression of complement factors of F2-juveniles was more affected by the grandmothers.

#### Epigenetic regulation genes (15 genes-total)

The global expression of five DNA-methylation genes, four histone de/methylation genes, and five genes responsible for acetylation and deacetylation of histone residues in one-week old F2-juveniles showed grandparental sex specific differences (PERMANOVA-*epigen*: F_3,174_ = 6.63, *p* = 0.035, Table 1, Fig. 2e). Multivariate pairwise comparisons displayed a stronger grand-paternal than grand-maternal effect over the 15 epigenetic regulation genes since grand-paternal and grand-biparental groups are not significantly different from each other (ANOSIM-*epigen*: F0-Bi vs. F0-Pat *p* = 0.396 Table 2﻿, Additional file 1: Table S2), displaying overlapping centers of gravity (Fig. 2e). A significant F0-sex x F2-bacteria interaction for all epigenetic genes (PERMANOVA-*epigen*: F_6,174_ = 1.22, *p* = 0.029, Table 1) further indicates grandparental sex-specific effects apart from the F2-bacteria treatment (ANOSIM-*epigen*: F0-Mat x F2-bacteria (V+ or T+) vs F0-Pat x F2-bacteria (V+ or T+) *p* < 0.004, Table 2).

#### Epigenetic regulation genes (individual categories)

The grandparental sex-specific immunological exposure primarily affected the expression of histone-acetylation genes (PERMANOVA-*hist.acetyl*: F_3,174_ = 12.47, *p* = 0.035, Table 1, Fig. 2f), while on the contrary a separate consideration of five DNA-methylation genes (PERMANVA-*DNA.methyl*: F_3,174_ = 6.09, *p* = 0.061, Table 1), four histone de/methylation genes (PERMANVA-*DNA.methyl*: F_3,174_ = 4.16, *p* = 0.195, Table 1), and three histone deacetylation genes (PERMANVA-*hist.deacetyl*: F_3,174_ = 5.65, *p* = 0.079, Table 1) were not significantly affected.

However, the combined expression of *Histone acetyltransferase KAT2A (BROMO)* and *Histone acetyltransferase HAT1 (MYST)* revealed pure grand-paternal influences, as the grand-maternal treatment group was not significantly different from the F0-naïve group but sets apart from the gran﻿d-biparental and grand-paternal treatments (ANOSIM-*hist.acetyl*: F0-Mat vs. F0-N *p* = 0.132; F0-Bi vs. F0-Mat *p* = 0.008; F0-Pat vs. F0-Mat *p﻿* = 0.001, Table 2, Additional file 1: Table S2, Fig. 2f).

### Grand-offspring treatment effect (F2-bacteria treatment)

The acute immune challenge of F2-offspring (grand-offspring treatment) with either *Vibrio* or *Tenacibaculum* bacteria significantly affected the multivariate expression of total 29 immune genes (PERMANOVA-*immune*: F_2,174_ = 3.08, *p* < 0.001, Table 1; ANOSIM-*immune:* F2-V+ vs. F2-N *p* = 0.022; F2-T+ vs. F2-N *p* = 0.021, Table 2) and total 15 epigenetic genes (PERMANOVA-*epigen*: F_2,174_ = 1.64, *p* = 0.030, Table 1; ANOSIM-*epigen:* F2-V+ vs. F2-N *p* = 0.005; F2-T+ vs. F2-N *p* = 0.049, Table 2). Further, we examined the effects of the acute immune challenge in the functional gene categories separately. An induced expression of innate immune genes (PERMANOVA-*innate*: F_2,174_ = 2.01, *p* < 0.026, Table 1), complement component genes (PERMANOVA-*complement*: F_2,174_ = 3.66, *p* = 0.001, Table 1) and genes involved in both innate & adaptive immune system (PERMANOVA-*innate&adaptive:* F_2,174_ = 5.47, *p* < 0.001, Table 1) was recorded. Expression of genes associated with DNA methylation processes (PERMANOVA-*DNA.methyl*, F_2,174_ = 2.26, *p* = 0.022, Table 1) and histone acetylation (PERMANOVA-*hist.acetylation*, F_2,174_ = 2.03, *p* = 0.019, Table 1) in F2-offspring was likewise significantly influenced upon the direct bacteria challenge. In contrast, genes of the adaptive immune system, histone de/methylation genes and histone deacetylation genes stayed unaffected (Table 1).

### Gene contribution

#### Immune gene expression (29 genes-total)

In the factor map the arrows of 29 immune genes were generally all pointing into the direction of the first principle component (Fig. 3a), which explains 30% of the total variation and visualizes in the corresponding PCA plots the grandparental treatment effect (Fig. 2a). Important genes with an average contribution above 60% were the innate immune genes *Calreticulin, Transferrin,* and *Natural resistance-associated macrophage protein (Nramp),* the adaptive immune genes *Integrin, HIVEP2,* and *HIVEP3* as well as *C﻿omplement component 3* and *9* (Fig. 3a)*.* Most of the analyzed innate immune genes showed a contribution between 40-60% such as *Peptidoglycan recognition protein, Heat shock protein 60 (Hsp60), Coagulation factor II, Lectin protein II, Kinesin, Allograft inflammation factor, Tyroproteinkinase, Ik-cytokine, Interferon,* and *Translocator protein (TSPO)* (Fig. 3a)*.* Besides, also following adaptive immune genes revealed a contribution between 40–60%: *CD45, Tapasin (TAP), Lymphocyte cytosolic protein 2, Lymphocyte antigen 75,* and *Immunoglobulin light chain* (Fig. 3a). Finally, *Chemokine 7, LPS induced TNFα factor, Complement component 1, Interleukin 10,* and *Interleukin 8* contributed below 40% of the average variance (Fig. 3a).

#### Epigenetic regulation genes (15 genes-total)

Epigenetic regulation genes with the highest average contribution of over 80% were *DNA(cytosine-5)-methyltransferases 3a* and *3b* (Fig. 3B), both responsible for *de novo* transfer of methyl groups to specific CpG sites in the DNA, permitting the formation of new methylation marks on unmethylated DNA [56–58]. Further, Histone acetyltransferase KAT2A (BROMO) which promotes acetylation of core histone proteins and with it transcriptional activation revealed over 80% of inertia contribution (Fig. 3b).

These important key genes were followed with 60–80% of contribution by the maintenance DNA methyltransferase *DNMT1* (Fig. 3b), which copies complementary marks of newly-replicated DNA by recognizing the hemimethylated sequences inherited from daughter strands [57].

Histone modification genes responsible for gene silencing or deactivation of gene transcription such as *Histone deacetylase 3 (HDAC3)* (60–80%), *Lysine specific demethylase (No66)* (60–80%) followed by *Histone deacetylase 1* (40–60%) and *Histone deacetylase 6* (40–60%) showed an intermediate contribution (Fig. 3b). The same pattern was true for genes promoting gene activation *Histone methyltransferase (ASH2)* (60–80%)*, Histone acetyltransferase HAT1 (MYST)* (40–60%), and *Transcription factor 8* (60–80%). Histone demethylation genes *Lysine specific demethylase 5B (JmjcPhD), Lysine specific demethylase 6A (TPR),* and DNA-methylation gene *N6admet-methyltransferase (N6admet)* contributed below 40% of the total variance (Fig. 3b).

## Discussion

### Grandparental immune priming effect

The parental impact on offspring immune system persisted, once the adaptive immune system reached maturation [41] and even continued into the second generation, affecting immune dynamics of grandoffspring in the pipefish *Syngnathus typhle*. Grandparental immunological treatment with heat-killed bacteria epitopes altered the gene expression patterns in the grandoffspring, affecting the global expression of 29 immune genes. The patterns are cross-correlated with the targeted functional gene groups, including genes of both innate and adaptive immune defense and complement component system. Upon immune challenge in the F2-generation, grandoffspring only induced immune gene expression if their grandparents already had experienced the bacterial epitopes. This result highlights the existence and importance of multigenerational TGIP, as only animals whose ancestors were exposed to a parasitic assemblage are able to quickly react towards an encounter with matching pathogens.

Genes of the innate immune system and complement component system were pre-dominantly affected, as they are essential in the pro-inflammatory response initiated 20 h after the bacterial injection. Central innate immune genes (>60% contribution) involved in driving this remarkable grandparental bacteria treatment effect were *Calreticulin, Transferrin, Natural resistance-associated macrophage protein (Nramp), Complement component 3* and *Complement component* 9*.* The latter two genes are key players in the alternative pathway of the complement component system, necessary for the immediate immune defense against invaders [59]. *Complement component 3* proteins recognize and tag bacteria and thereby activate the lytic pathway during which the membrane attack complex (MAC) is formed under the support of *Complement component 9* [59]. Activation proteins such as *Natural resistance-associated macrophage protein* trigger macrophages activity that perform phagocytosis and remove pathogens with the production of reactive oxygen species (respiratory burst) and a release of hydrolytic lysozymes [60], while *Calreticulin* chaperones assist in promoting the phagocytosis process and clearance of apoptotic cells. A primitive but effective antimicrobial mechanism of the innate immune system du﻿ring which actively nutriens (iron) are removed from bacterial pathogens, a process referred to as iron-withholding, is maintained by *Transferrin* and also intracellularly within the phagosome over *Natural resistance-associated macrophage proteins* [61, 62]. Hence, the first line of innate immune defense is activated upon the grandparental treatment in the juvenile pipefish [63]. Further innate immune genes with a lower average contribution (between 40-60%) are also involved in immediately available and inducible pathways like bacteria recognition (*C-type lectin II, Peptidoglycan recognition protein, Lectin protein II)*, antiviral response (*Interferon*), oxidative burst by macrophages (*Translocator protein),* stress response over molecular chaperone (*Heat shock protein 60*) as well as general inflammatory response (*Allograft inflammation factor, Coagulation factor II, Kinesin, Tyroproteinkinase, Ik-cytokine*) [64, 65]. The assessed adaptive immune genes were not affected by the F0-bacteria treatment, potentially due to the on-going maturation of the adaptive immune system in one-week-old pipefish [66] and/or the rather early time point of sampling (20 h after injection). Yet*,* our former data implys that the expression of the same immune genes used here positively correlates with an activation of the innate (monocytes) and adaptive (lymphocytes) cellular immune system [19, 40, 67], suggesting that induced gene expression is linked to a physiological impact of TGIP.

### Grandparental sex-specific effect (grand-maternal vs. grand-paternal effects)

Grandfathers and grandmothers might have evolved different strategies to achieve an optimal immunological protection of their grandoffspring. Here, we investigated complementing grandparental sex-specific contribution to different pathways of the immune system in one-week-old F2-juveniles. Grand-paternal immunological experience boosted the activity of the innate immune system in their grandoffspring. This male-specific effect on innate immunity is consistent with the result of TGIP over one generation [40]. Grandfathers transfer immediate protection via innate immunity during male pregnancy against prevalent pathogens of their surrounding environment.

In contrast, grandmothers largely influenced genes of the complement component system. In teleosts mothers activate the complement system of their offspring via the deposition of a variety of diverse complement component proteins such as C1, C3, a﻿nd C4 into the eggs [54, 68]. Grandoffspring might even profit from mRNA transcripts that can likewise be transferred into the egg yolk [26]. A grand-maternal priming of the complement system can result in an earlier usage of acquired immune responses as it supports the antibody-mediated adaptive immune response. This implies that a stronger response against pathogens and parasites that linger in the environment over several generations is initiated. Since grandparents differentially influence the distinct immune pathways of the F2-offspring, grand-maternal and grand-paternal immune priming can complement each other leading to a balanced effect on total expression of 29 immune genes. Parental sex - specific influences on different immune pathway observed in former results of the F1-generation [40] lasted into the F2-generation and reflect an efficient strategy to maintain optimal protection against parasites by both parents correspondingly both grandparents.

Our data now suggest that information on prevalent bacterial epitopes is conserved and sex-specifically transferred, leading to complementing biparental TGIP over two generations. With this strategy parents not only deliver specific protection to their genetic offspring and grandoffspring, but they also transfer the opportunity to plastically adapt to the prevailing pathogen environment. In contrast to most species with conventional sex roles, in a sex-role reversed pipefish offspring are born into the paternal environment and, thus, share the paternal parasitic experience. This makes the transfer of immunity via the paternal line likely to be adaptive. Hence, both fathers and grandfathers will increase their fitness by altering their phenotype to optimally acclimatize offspring to the local parasitic environment [38]. Yet, female specific immune priming effects still remained, to a lower extent. Potentially this ancient evolution of beneficial maternal transfer of immune components into the eggs was still selected for, as a certain likelihood of matching parasite environment in their seasonal habitat (seagrass meadows) remained. When low dispersal might have resulted in habitat matching between grand-maternal and grandoffspring environment, maternal transfer of immunity should have been selected [22]. While most species boost their offspring immune response exclusively via maternal TGIP, pipefish rely on both, on maternal and paternal TGIP [41] that last for at least two generations. This dual developmental plasticity with sex-specific effector pathways gives the next generations an evolutionary advantage in reacting towards potentially virulent parasites. The grand-biparental TGIP is adaptive, provided that the maternal and paternal parasitic environment is experienced by their offspring correspondingly grandoffspring [22]. TGIP, accordingly, gives individuals an advantage whose ancestors successfully defended parasites and transfer this experience to the following generations.

Such heightened reaction to a pathogen should only be expressed under certain ecological circumstances as strong expression of immunity and the maintenance of inducible defense is costly due to high energy demand [69]. Bi-parental immune priming is traded off with delayed maturation time of adult F1-males, reduced fecundity and reproduction of the adult F1-generation, in case of parental bacteria exposure, indicating a compensational effect of reduced energy investment into reproduction [40, 41]. These costs might constrain the overall beneficial net output of biparental immune priming [40, 41]. Nevertheless, selection for grandparental immune priming designates that adaptive net influence and total benefits outweighed the associated costs.

### Mechanism of immune priming (epigenetic regulation)

Our results cannot be explained by parasite-induced selection, as we used virulent heat-killed bacteria for the immune challenges. This presumes that the inheritance mechanism is non-DNA sequence based. The mechanisms permitting immunological information to be preserved via the paternal line over two generations most likely rely on a combination of small soluble immune components and epigenetic factors that are transferred via the sperm, the placenta-like structure or the fluid of the paternal brood-pouch tissue. As innate immune genes were predominantly influenced by the grand-paternal treatment, these genes might play a crucial role in the paternal transmission process.

DNA methylation and histone modifications are responsible for regulating packing and de-packing of the chromatin structure around histone molecules [70] and, consequently, the activation or deactivation of transcription processes for our targeted immune genes. That such epigenetic modifications of the genome can be responsible for paternal effects was recently demonstrated in zebrafish displaying paternal methylome transmission [34, 37]. In our study, the expression of total 15 genes connected to DNA-methylation and histone de/methylation and de/acetylation in one-week-old F2 juveniles showed a significant change of expression in case of grandparental bacteria exposure. Epigenetic regulation genes that displayed a high contribution with over 80% were *DNA-methyltransferase 3a*, *DNA-methyltransferase 3b*. As *de novo* methylation via DNMT3a/b causes new chemical modifications of the DNA [56, 58] and is essential for maternal and paternal imprinting [71]*,* DNMT3a/b are potentially crucial mediators for epigenetic changes based on environmental stressors. In accordance to previous findings epigenetic regulation genes might not only be central regulators of parental immune priming [40, 41], but also of grandparental immune priming, revealing a persisting effect into the second generation. In addition, we found strong evidence that histone acetylation genes (*Histone acetyltransferase KAT2A (BROMO)* and *Histone acetyltransferase HAT1 (MYST))* regulating positively the accessibility of the DNA sequence for transcription processes by addition of acetyl groups to histone tails [72] were strongly influenced by the grandfathers solely. Histone modifications are supposed to be heritable across generations [73–75] and might carry epigenetic information [76]*.* The reaction to repeated pathogen exposure in macrophages involves positive histone marks and chromatin remodeling at specific promotors [77]. Moreover, it was suggested that histone modifications are associated with immune memory following a viral infection in CD8  T-cells [78]. The recent findings of parental [40] and grandpaternal influences on histone modification genes upon bacterial immune challenge suggests that the regulation of immune priming might be mediated with heritable marks stored on histones.

Our data, thus, propose that environmental stressors like pathogens leave an epigenetic mark on the genome affecting gene expression of genes associated with the immune system and transcriptional regulation that can be inherited over multiple generations. The fact that grandparental TGIP involves epigenetic mechanisms may result in a novel selection scenario for the evolution of TGIP along the paternal line, as the argument that male sperm is too small to transfer any more than just the DNA does not apply any longer. The sperm is thus potentially not only a sole messenger of “the other half of the offspring DNA”, but also an important mediator for developmental plasticity and fast phenotypic acclimation to environmental changes [2, 32, 38, 70].

## Conclusions

Transgenerational effects on immunity in vertebrates are not short-term but can be sustained across two consecutive generations by the involvement of epigenetic regulation mechanism. These grandparental immune priming effects in the pipefish revealed complementing sex-specific contribution to different pathways of their grandoffspring immune system. Although TGIP might be beneficial on the individual level, it also involves ecological and evolutionary consequences on population level and has the potential to change disease dynamics and the spread of epidemics in a population [79, 80]. Under negative-frequency dependent selection, rare parasite alleles may spread quickly in a population, while it takes time for the hosts as a population to counter-adapt under a Red-Queen dynamic [81]. In contrast, as an individual response, TGIP plays out within one generation, because the exposure to a novel parasite will already be met with an amplified immune reaction in the next generation. With this, the advantage of the novel parasite genotypes vanishes. This dampens the amplitude of predicted frequency dependent selection and may slow down Red Queen dynamics, giving the host an advantage in fast clearance of novel pathogen genotypes [79, 80].

According to neutral genetic markers, *Syngnathus typhle* from different sites in the Baltic Sea all belong to the same population [82]. This is in line with the broad-nosed pipefish migratory behaviour: from open waters in winter to shallow seagrass meadows along the coastline in summer to exert mating and reproduction [83]. While in this sex-role reversed species, females display secondary sexual signals and are subject to multiple mating, males as the choosing sex are bound to their offspring during pregnancy. With respect to the seasonal migration pattern and the larval exposure to the pathogenic environment that their fathers already experienced, the investment into grandparental immune priming can be adaptive as individuals will be pre-adapted for the pathogen fauna in which the subsequent generations mate and release their offspring. On-going climate change with higher temperatures and lower salinity levels induces the abundance and virulence of pathogenic strains e.g. *Vibrio* particularly during the summer season [84]. The efficient transfer of immunological information about prevalent pathogenic threads is key for efficient short-term acclimation to changing virulence patterns [84] with benefits occurring particularly during the summer mating season in highly exposed shallow seagrass meadows. Future work should focus on the evaluation of resistance effects in a survival experiment, bacterial specificity processes, and an in depth analysis of the physiological mechanisms mediating grandparental TGIP.

## Methods

### Grandparental generation (F0-treatment)

Broad-nosed pipefish *Syngnathus typhle* were caught in the south-western Baltic Sea (54°44‘N; 9°53’E, Germany) in spring 2013 and acclimatized to local summer conditions (15psu, 18 °C, 14:10 h light:dark) within three weeks. We hosted the pipefish in local water out of Kiel Fjord, which was initially cleaned by a sand filter followed by 5, 20 and 50 μm mesh filter, UV-light, surface skimmer and biological filter to reduce the amount of microbes in the aquaria system. The parental generation (F0) was vaccinated as described previously [40], using a combination of heat-killed immunological novel *Vibrio* spp*.* and *Tenacibaculum maritimum* bacteria (F0-bacteria) or were left naïve (F0-N) as control. Immune-challenged mature pipefish were used in following mating design: 1. Control: [♀F0-naïve x ♂F0﻿-naïve]; 2. Paternal: [♀F0-naïve x ♂F0-bacteria]; 3. Maternal: [♀F0-bacteria x ♂F0-naïve] and 4. Biparental: [♀F0-bacteria x ♂F0-bacteria] and kept according to their mating pairs (families) in separate 36×80 L semi-flow through aquaria (16 family replicates per parental bacteria treatment and eight per control group; 56 families; Fig. 1). For the immune challenge, we used a combination of two distinct marine bacteria species to cover a potential wide range of immunological pathways, which could be differentially influenced by TGIP. The *Vibrio* spp. bacteria used in this experiment were an isolate of an Italian pipefish, allopatric and novel for the Baltic pipefish species [85]. The *Tenacibaculum maritinum* bacteria were an isolate of a pacific seabream species of Japan [86], and have, to our knowledge, not been in contact with the Baltic pipefish before. Both, *Vibrio* (s-shaped and flagellated) and *Tenacibaculum* (rod-shaped but non-flagellated), are common gram-negative marine bacteria causing the following diseases in teleost. *Tenacibaculum maritimum* induces *‘Flexibacteriosis﻿’* also known as ‘black patch necrosis’ in marine fish [87, 88]. This disease is mainly characterised by haemorrhagic skin lesions, an ulcerative condition leading to important mortalities among marine fish species [87, 88]. *Vibrio* bacteria can trigger *‘Vibriosis’,* a systemic disease of marine fishes [89], and e.g. *Vibrio harveyi* species are known to cause mass mortalities in captive bred seahorses [90]. The combination of *Vibrio* and *Tenacibaculum* permitted to cover an extended range of bacteria specific TGIP [41]*.*

### Filial generation 1 (F1-treatment)

F1-offspring were reared in 36×80 L aquaria and stayed separated in their tanks according to their parental treatment. Depending on their developmental stage, fish were fed with *Artemia salina* naupliae, copepods (*Acarcia spec*) and mysids (*Mysis spec*). F1-individuals were crossed within former parental treatment groups but left immunologically naïve (from each of the 4 parental treatment groups five families were chosen to do F1-crosses, resulting in 20 F1-families).

### Filial generation 2 (F2-treatment)

In spring 2014, one-week old (post birth) F2-juveniles (F2) were exposed to the same heat-killed *Vibrio* (F2-V+) and *Tenacibaculum* (F2-T+) bacteria used for the F0-generation or left naïve (F2-N) (per F1-crossing four families produced F2-offspring resulting in 16 F1-families). Out of each family, 12 individuals were chosen for the direct immune challenge. Per F2-offspring treatment (F2-V+, F2-T+, F2-N) four individual replicates were used, resulting in a total of 192 samples. Upon immune challenge, F2-juveniles were kept for 20 h in 10×10 cm tanks at 18 °C and 15 psu, using one tank per F2-offspring treatment and family. After the incubation time, juvenile body standard length [cm] was measured and animals were killed with MS 222. The body was transferred into 1 ml RNA-later, kept at 4 °C for 24 h, and then frozen at −20 °C.

### Gene expression and data processing

We quantified the mRNA-level of 44 target genes and 4 housekeeping genes in 192 samples using quantitative real time PCR (qPCR) over a 96.96 dynamic array Fluidigm-BioMark™ system as described previously [40]. Thereby, the RNA extraction of 192 tissue samples and reverse transcription into cDNA was performed with a fixed amount of 800 [ng/μl] per sample as described previously [40].

For the following gene expression data analysisthe mean cycle time (Ct), standard deviation (SD), and the coefficient of variation (CV) were calculated. Samples with a CV larger than 4% were removed [91]. As the combination of the housekeeping genes ubiquitin (Ubi) and ribosome protein (Ribop) showed the highest stability (geNorm M > 0.85) [92], their geomean was used to quantify relative gene expression of each target gene by calculating − ∆Ct-values [41]. Multivariate statistics were used to infer differences in the entire expression pattern of 29 immune genes and 15 epigenetic regulation genes, for more detailed evaluation the genes were also divided into following functional gene categories: (i) innate immune system, (ii) adaptive immune system. (iii) innate and adaptive immune genes, (iv) complement system, (v) DNA methylation, (vi) histone de/methylation, (vii) histone deacetylation, and (vii) histone acetylation [40, 41].

### Multivariate statistics

Statistical multivariate tests and plots were performed in R v 3.2.2 [93] and PRIMERv6 [94]. Grandparental sex specific influences (‘F0-sex’) (defined by four levels I: F0-biparental bacteria treatment; II: F0-maternal bacteria treatment; III: F0-paternal bacteria treatment; IV: F0-naïve no bacteria treatment) were evaluated by using F0-sex as main factor and assessing its interaction with F2-bacteria treatment (‘F0-sex x F2-bacteria’). Consequently, we fitted a PERMANOVA model (‘vegan’ package - ‘adonis’ function in R) for each functional gene category (see last paragraph) based on an Euclidean distance matrix, by defining ‘F0-sex’ and ‘F2-bacteria’ treatments as fixed factors and stratifying permutations within each family replicate 10000 times (family was included as random factor). Standard length of F2-juveniles was included as covariate in the model to correct for the dependence between gene expression and body size. Significant PERMANOVA tests were followed by an ANOSIM (Analysis of Similarity) with the software PRIMERv6 [94] which allowed pairwise comparisons between the different levels of F0-sex and F2-bacteria treatment groups as well as their interaction in a multivariate approach [95]. The ANOSIM was conducted likewise with an Euclidean distance matrix and 10000 permutations.

Principle component analysis (PCA) for graphical visualization was carried out based on an Euclidean distance matrix with the ‘ade4’ package in R [96]. For drawing the PCAs, we implemented the first three axes to obtain a projection of the whole data set onto a conveniently small dimension and to assess the clustering according to the F0-bacteria treatment due to differential gene expression. PCAs were solely performed for functional gene categories that revealed a significant F0-sex effect (Fig. 2). In addition, we evaluated the percentage of contribution of response variables (genes) in explaining the variations retained by the principle components (PCs) by applying the ‘factoextra’ package implemented in R [97]. The total contribution of a variable (gene) which explains the variations elicited by the principle components (PCs) was calculated within the function ‘fvizcontrib’ [97]. Gene contribution (%) was visualized using a factor map in which a implemented colour gradient highlights most important genes with the highest contribution of variance (Fig. 3) [97].

## Additional file

Additional file 1: Table S1. Additional values of 2-way PERMANOVA output. Multivariate PERMANOVA analysis to assess the effect and interaction of two fixed factors F0-sex and F2-bacteria while including size as covariate and family as strata term on relative gene expression data (−∆Ct-values). Each analysis was based on an Euclidean distance matrix with* p*-values obtained by 10000 permutations. Significant *p*-values are marked in bold letters and asterix symbol (significance code: <0.001***, 0.001**, 0.01*, 0.1 > *p*-value ≥ 0.05 trend ●). R^2^ value indicate the percentage of variance explained by the model. **Table S2**. Results from PERMANOVA and ANOSIM analysis of one-week-old F2-juveniles per functional gene categories. Multivariate ANOSIM was performed following significant PERMANOVA effects to assess differences in the gene expression profiles per treatment groups applying pairwise comparison on relative gene expression data (−∆Ct-values) based on a Euclidean distance matrix and 10000 permutations. Pairwise comparison was conducted for following fixed factors and their interactions: F0-sex (grandparental (F0-Bi), grand-maternal (F0-Mat), grand-paternal (F0-Pat), grandparental control (F0-N)) and F2-bacteria (F2-bacteria control (F2-N), F2-bacteria *Vibrio* (F2-V+) and F2-bacteria *Tenacibaculum* (F2-T+)).(DOCX 56 kb)
