# Supplementary material for: Grandparental immune priming in the pipefish Syngnathus typhle
Source: BMC Evol Biol. 2017 Feb 7;17:44. doi: 10.1186/s12862-017-0885-3 (PMC5297188; doi:10.1186/s12862-017-0885-3)
Supplement: Additional file 1: Table S1. — Additional values of 2-way PERMANOVA output. Multivariate PERMANOVA analysis to assess the effect and interaction of two fixed factors F0-sex and F2-bacteria while including size as covariate and family as strata term on relative gene expression data (−∆Ct-values). Each analysis was based on an Euclidean distance matrix with p-values obtained by 10000 permutations. Significant p-values are marked in bold letters and asterix symbol (significance code: <0.001***, 0.001**, 0.01*, 0.1 > p-value ≥ 0.05 trend ●). R2 value indicate the percentage of variance explained by the model. Table S2. Results from PERMANOVA and ANOSIM analysis of one-week-old F2-juveniles per functional gene categories. Multivariate ANOSIM was performed following significant PERMANOVA effects to assess differences in the gene expression profiles per treatment groups applying pairwise comparison on relative gene expression data (−∆Ct-values) based on a Euclidean distance matrix and 10000 permutations. Pairwise comparison was conducted for following fixed factors and their interactions: F0-sex (grandparental (F0-Bi), grand-maternal (F0-Mat), grand-paternal (F0-Pat), grandparental control (F0-N)) and F2-bacteria (F2-bacteria control (F2-N), F2-bacteria Vibrio (F2-V+) and F2-bacteria Tenacibaculum (F2-T+)).(DOCX 56 kb) [file 12862_2017_885_MOESM1_ESM.docx]

## Additional file

**Table S1: Additional values of 2-way PERMANOVA output.** Multivariate PERMANOVA analysis to assess the effect and interaction of the two fixed factors F0-sex and F2-F2-bacteria while including Size as covariate and family as strata term on relative gene expression values (−∆Ct-values). Each analysis was based on an Euclidean distance matrix with p-values obtained by10000 permutations. Significant p-values are marked in bold letters and asterix (significance code: <0.001***, 0.001**, 0.01*, 0.1>p-value≥0.05 trend ●). R^2^ value indicate the percentage of variance explained by the model.

|  | **Df** | **SumsOfSqs** | **MeanSqs** | **F.Model** | **R2** | **Pr(>F)** |  |
| --- | --- | --- | --- | --- | --- | --- | --- |
| **Immune genes (29)** | | | | | | | |
| F0-sex | 3 | 828.3 | 276.114 | 6.8205 | 0.09759 | **0.0002** | ******* |
| F2-bacteria | 2 | 249.5 | 124.726 | 3.0809 | 0.02939 | **1.00E-04** | ******* |
| Size | 1 | 45.9 | 45.882 | 1.1334 | 0.00541 | 0.640936 |  |
| F0-sex:F2-bacteria | 6 | 320 | 53.331 | 1.3174 | 0.0377 | **0.008999** | ****** |
| Residuals | 174 | 7044.1 | 40.483 |  | 0.82991 |  |  |
| Total | 186 | 8487.7 |  |  | 1 |  |  |
| **Innate genes (13)** | | | | | | | |
| F0-sex | 3 | 397.3 | 132.431 | 6.6722 | 0.09509 | **0.0037** | ****** |
| F2-bacteria | 2 | 79.7 | 39.859 | 2.0082 | 0.01908 | **0.026397** | ***** |
| Size | 1 | 25.1 | 25.054 | 1.2623 | 0.006 | 0.431157 |  |
| F0-sex:F2-bacteria | 6 | 222.3 | 37.046 | 1.8664 | 0.0532 | **0.007499** | ****** |
| Residuals | 174 | 3453.6 | 19.848 |  | 0.82663 |  |  |
| Total | 186 | 4177.9 |  |  | 1 |  |  |
| **Adaptive genes (8)** | | | | | | | |
| F0-sex | 3 | 152.59 | 50.865 | 11.5274 | 0.1585 | 0.1076 |  |
| F2-bacteria | 2 | 8.78 | 4.39 | 0.995 | 0.00912 | 0.1841 |  |
| Size | 1 | 7.52 | 7.524 | 1.7052 | 0.00782 | 0.521 |  |
| F0-sex:F2-bacteria | 6 | 26.09 | 4.348 | 0.9853 | 0.0271 | 0.1001 |  |
| Residuals | 174 | 767.78 | 4.413 |  | 0.79747 |  |  |
| Total | 186 | 962.76 |  |  | 1 |  |  |
| **Innate & Adaptive genes (5)** | | | | | | | |
| F0-sex | 3 | 205.08 | 68.36 | 5.8844 | 0.08498 | **0.0012** | ****** |
| F2-bacteria | 2 | 127.2 | 63.601 | 5.4747 | 0.05271 | **1.00E-04** | ******* |
| Size | 1 | 10.04 | 10.04 | 0.8642 | 0.00416 | 0.4604 |  |
| F0-sex:F2-bacteria | 6 | 49.63 | 8.272 | 0.712 | 0.02056 | 0.6221 |  |
| Residuals | 174 | 2021.38 | 11.617 |  | 0.83759 |  |  |
| Total | 186 | 2413.34 |  |  | 1 |  |  |
| **Complement component genes (3)** | | | | | | | |
| F0-sex | 3 | 73.37 | 24.4576 | 5.311 | 0.07859 | **0.0171983** | ***** |
| F2-bacteria | 2 | 33.75 | 16.8759 | 3.6646 | 0.03615 | **0.0008999** | ******* |
| Size | 1 | 3.26 | 3.2633 | 0.7086 | 0.0035 | 0.790221 |  |
| F0-sex:F2-bacteria | 6 | 21.99 | 3.6654 | 0.7959 | 0.02355 | 0.2372763 |  |
| Residuals | 174 | 801.29 | 4.6051 |  | 0.85822 |  |  |
| Total | 186 | 933.67 |  |  | 1 |  |  |
| **Epigenetic genes (15)** | | | | | | | |
| F0-sex | 3 | 65.3 | 21.7673 | 6.627 | 0.09692 | **0.0346** | ***** |
| F2-bacteria | 2 | 10.76 | 5.381 | 1.6382 | 0.01597 | **0.0303** | ***** |
| Size | 1 | 2.05 | 2.052 | 0.6247 | 0.00305 | 0.8942 |  |
| F0-sex:F2-bacteria | 6 | 24.12 | 4.0195 | 1.2237 | 0.03579 | **0.0291** | ***** |
| Residuals | 174 | 571.53 | 3.2847 |  | 0.84827 |  |  |
| Total | 186 | 673.76 |  |  | 1 |  |  |
| **DNA-methylation genes (5)** | | | | | | | |
| F0-sex | 3 | 24.898 | 8.2994 | 6.0937 | 0.0893 | 0.06149 | . |
| F2-bacteria | 2 | 6.168 | 3.0839 | 2.2643 | 0.02212 | **0.022** | ***** |
| Size | 1 | 1.14 | 1.1404 | 0.8373 | 0.00409 | 0.81152 |  |
| F0-sex:F2-bacteria | 6 | 9.632 | 1.6053 | 1.1787 | 0.03455 | 0.08149 | . |
| Residuals | 174 | 236.983 | 1.362 |  | 0.84995 |  |  |
| Total | 186 | 278.821 |  |  | 1 |  |  |
| **Histone-de/methylation genes (4)** | | | | | | | |
| F0-sex | 3 | 10.304 | 3.4347 | 4.1582 | 0.06364 | 0.19498 |  |
| F2-bacteria | 2 | 1.122 | 0.5609 | 0.679 | 0.00693 | 0.51575 |  |
| Size | 1 | 0.168 | 0.168 | 0.2033 | 0.00104 | 0.84382 |  |
| F0-sex:F2-bacteria | 6 | 6.603 | 1.1005 | 1.3323 | 0.04078 | 0.08189 | . |
| Residuals | 174 | 143.726 | 0.826 |  | 0.88762 |  |  |
| Total | 186 | 161.923 |  |  | 1 |  |  |
| **Histone deacetlyation genes (3)** | | | | | | | |
| F0-sex | 3 | 7.071 | 2.35709 | 5.65 | 0.08387 | 0.07909 | . |
| F2-bacteria | 2 | 1.027 | 0.51374 | 1.2315 | 0.01219 | 0.12559 |  |
| Size | 1 | 0.579 | 0.57873 | 1.3872 | 0.00686 | 0.62064 |  |
| F0-sex:F2-bacteria | 6 | 3.041 | 0.5068 | 1.2148 | 0.03607 | 0.0599 | . |
| Residuals | 174 | 72.59 | 0.41718 |  | 0.86101 |  |  |
| Total | 186 | 84.308 |  |  | 1 |  |  |
| **Histone acetylation genes (2)** | | | | | | | |
| F0-sex | 3 | 19.588 | 6.5292 | 12.4683 | 0.16835 | **0.0354** | ***** |
| F2-bacteria | 2 | 2.128 | 1.0639 | 2.0317 | 0.01829 | **0.0186** | ***** |
| Size | 1 | 0.08 | 0.08 | 0.1528 | 0.00069 | 0.89561 |  |
| F0-sex:F2-bacteria | 6 | 3.437 | 0.5728 | 1.0939 | 0.02954 | 0.06499 | . |
| Residuals | 174 | 91.117 | 0.5237 |  | 0.78313 |  |  |
| Total | 186 | 116.349 |  |  | 1 |  |  |

**Table S2: Results from PERMANOVA and ANOSIM analysis of one-week-old F2-juveniles per functional gene categories.** Multivariate ANOSIM was performed following significant PERMANOVA effects to assess differences in the gene expression profiles per treatment groups applying pairwise comparison on relative gene expression data (−∆Ct-values) based on a Euclidean distance matrix and 999 permutations. Pairwise comparison was conducted for following fixed factors and their interactions: F0-bacteria treatment effect (grandparental control (F0-N) versus grandparental bacteria (F0-bacteria) (*Vibrio* & *Tenacibaculum*)), `F0-sex´ (grandparental (F0-Bi), grand-maternal (F0-Mat), grand-paternal (F0-Pat), grandparental control (F0-N)) and `F2-bacteria´ (F2-F2-bacteria control (F2-N), F2-F2-bacteria *Vibrio* (F2-V+) and *Tenacibaculum* (F2-T+).

| **F2-juveniles (One-week-old)** | **Immune genes [29 total]** | **Innate genes [13]** | **Adaptive genes [8]** | **Innate & Adaptive genes [5]** | **Complement component genes [3]** | **Epigenetic genes**  **[15 total]** | **DNA-methylation genes [5]** | **Histone-de/methylation genes [4]** | **Histone deacetlyation genes [3]** | **Histone acetylation genes [2]** |
| --- | --- | --- | --- | --- | --- | --- | --- | --- | --- | --- |
|  |  |  |  |  |  |  |  |  |  |  |
| **F0-sex (DF=3)** | **<0.001***** | **0.004**** | *ns* | **0.001**** | **0.017*** | **0.035*** | *ns* | *ns* | *ns* | **0.035*** |
| **ANOSIM-Global R** | 0.115 | 0.12 | *ns* | 0.104 | 0.054 | 0.088 | *ns* | *ns* | *ns* | 0.088 |
| **Significance level** | 0.1% | 0.1% | *ns* | 0.1% | 0.1% | 0.1% | *ns* | *ns* | *ns* | 0.1% |
|  |  |  |  |  |  |  |  |  |  |  |
| **F0-Bi, F0-Mat** | **0.004** | **0.001** | *ns* | **0.003** | 0.168 | **0.027** | *ns* | *ns* | *ns* | **0.008** |
| **F0-Bi, F0-Pat** | **0.003** | 0.096 | *ns* | **0.002** | **0.009** | 0.396 | *ns* | *ns* | *ns* | 0.384 |
| **F0-Mat, F0-Pat** | **0.007** | **0.001** | *ns* | **0.001** | 0.202 | **0.001** | *ns* | *ns* | *ns* | **0.001** |
| **F0-Bi, F0-N** | **0.001** | **0.001** | *ns* | **0.001** | **0.002** | **0.001** | *ns* | *ns* | *ns* | **0.009** |
| **F0-Mat, F0-N** | **0.001** | **0.001** | *ns* | **0.002** | **0.002** | **0.009** | *ns* | *ns* | *ns* | 0.132 |
| **F0-Pat, F0-N** | **0.001** | **0.001** | *ns* | **0.001** | 0.124 | **0.001** | *ns* | *ns* | *ns* | **0.007** |
|  |  |  |  |  |  |  |  |  |  |  |
| **F2-Bacteria (DF=2)** | **<0.001***** | **0.026*** | *ns* | **<0.001***** | **0.001***** | **0.030*** | **0.022*** | *ns* | *ns* | **0.019*** |
| **ANOSIM-Global R** | 0.022 | 0.018 | *ns* | 0.026 | 0.024 | 0.004 | 0.011 | *ns* | *ns* | 0.009 |
| **Significance level** | 7.4% | 11.5% | *ns* | 2.1% | 5.9% | 31.9% | 20.9% | *ns* | *ns* | 72.9% |
|  |  |  |  |  |  |  |  |  |  |  |
| **F2-V+, F2-T+** | *ns* | *ns* | *ns* | *ns* | *ns* | *ns* | *ns* | *ns* | *ns* | *ns* |
| **F2-V+, F2-N** | **0.022** | **0.011** | *ns* | **0.006** | **0.024** | **0.005** | **0.005** | *ns* | *ns* | *ns* |
| **F2-T+, F2-N** | **0.021** | **0.016** | *ns* | **0.030** | *ns* | **0.049** | **0.049** | *ns* | *ns* | **0.05** |
|  |  |  |  |  |  |  |  |  |  |  |
| **F0-sex x F2-Bacteria (DF=6)** | **0.009**** | **0.007**** | *ns* | *ns* | *ns* | **0.029*** | *ns* | *ns* | *ns* | *ns* |
| **ANOSIM-Global R** | 0.105 | 0.103 | *ns* | *ns* | *ns* | 0.074 | *ns* | *ns* | *ns* | *ns* |
| **Significance level** | 0.1% | 0.1% | *ns* | *ns* | *ns* | 0.1% | *ns* | *ns* | *ns* | *ns* |
|  |  |  |  |  |  |  |  |  |  |  |
| **F0-Bi/F2-V+, F0-Bi/F2-T+** | *ns* | *ns* | *ns* | *ns* | *ns* | *ns* | *ns* | *ns* | *ns* | *ns* |
| **F0-Bi/F2-V+, F0-Bi/F2-N** | *ns* | *ns* | *ns* | *ns* | *ns* | *ns* | *ns* | *ns* | *ns* | *ns* |
| **F0-Bi/F2-V+, F0-Mat/F2-V+** | *ns* | *ns* | *ns* | *ns* | *ns* | *ns* | *ns* | *ns* | *ns* | *ns* |
| **F0-Bi/F2-V+, F0-Mat/F2-T+** | *ns* | *ns* | *ns* | *ns* | *ns* | *ns* | *ns* | *ns* | *ns* | *ns* |
| **F0-Bi/F2-V+, F0-Mat/F2-N** | **0.03** | **0.022** | *ns* | *ns* | *ns* | *ns* | *ns* | *ns* | *ns* | *ns* |
| **F0-Bi/F2-V+, F0-Pat/F2-V+** | *ns* | *ns* | *ns* | *ns* | *ns* | *ns* | *ns* | *ns* | *ns* | *ns* |
| **F0-Bi/F2-V+, F0-Pat/F2-T+** | *ns* | *ns* | *ns* | *ns* | *ns* | *ns* | *ns* | *ns* | *ns* | *ns* |
| **F0-Bi/F2-V+, F0-Pat/F2-N** | *ns* | *ns* | *ns* | *ns* | *ns* | *ns* | *ns* | *ns* | *ns* | *ns* |
| **F0-Bi/F2-V+, F0-N/F2-V+** | **0.002** | **0.003** | *ns* | *ns* | *ns* | **0.01** | *ns* | *ns* | *ns* | *ns* |
| **F0-Bi/F2-V+, F0-N/F2-T+** | **0.011** | **0.012** | *ns* | *ns* | *ns* | **0.003** | *ns* | *ns* | *ns* | *ns* |
| **F0-Bi/F2-V+, F0-N/F2-N** | **0.008** | **0.006** | *ns* | *ns* | *ns* | **0.001** | *ns* | *ns* | *ns* | *ns* |
| **F0-Bi/F2-T+, F0-Bi/F2-N** | *ns* | *ns* | *ns* | *ns* | *ns* | *ns* | *ns* | *ns* | *ns* | *ns* |
| **F0-Bi/F2-T+, F0-Mat/F2-V+** | *ns* | *ns* | *ns* | *ns* | *ns* | **0.054** | *ns* | *ns* | *ns* | *ns* |
| **F0-Bi/F2-T+, F0-Mat/F2-T+** | *ns* | *ns* | *ns* | *ns* | *ns* | **0.05** | *ns* | *ns* | *ns* | *ns* |
| **F0-Bi/F2-T+, F0-Mat/F2-N** | *ns* | **0.015** | *ns* | *ns* | *ns* | **0.075** | *ns* | *ns* | *ns* | *ns* |
| **F0-Bi/F2-T+, F0-Pat/F2-V+** | *ns* | *ns* | *ns* | *ns* | *ns* | *ns* | *ns* | *ns* | *ns* | *ns* |
| **F0-Bi/F2-T+, F0-Pat/F2-T+** | *ns* | *ns* | *ns* | *ns* | *ns* | *ns* | *ns* | *ns* | *ns* | *ns* |
| **F0-Bi/F2-T+, F0-Pat/F2-N** | *ns* | *ns* | *ns* | *ns* | *ns* | *ns* | *ns* | *ns* | *ns* | *ns* |
| **F0-Bi/F2-T+, F0-N/F2-V+** | **0.002** | **0.003** | *ns* | *ns* | *ns* | **0.007** | *ns* | *ns* | *ns* | *ns* |
| **F0-Bi/F2-T+, F0-N/F2-T+** | **0.01** | **0.019** | *ns* | *ns* | *ns* | **0.001** | *ns* | *ns* | *ns* | *ns* |
| **F0-Bi/F2-T+, F0-N/F2-N** | **0.001** | **0.001** | *ns* | *ns* | *ns* | **0.001** | *ns* | *ns* | *ns* | *ns* |
| **F0-Bi/F2-N, F0-Mat/F2-V+** | **0.049** | **0.033** | *ns* | *ns* | *ns* | **0.021** | *ns* | *ns* | *ns* | *ns* |
| **F0-Bi/F2-N, F0-Mat/F2-T+** | **0.023** | **0.011** | *ns* | *ns* | *ns* | **0.006** | *ns* | *ns* | *ns* | *ns* |
| **F0-Bi/F2-N, F0-Mat/F2-N** | **0.025** | **0.009** | *ns* | *ns* | *ns* | *ns* | *ns* | *ns* | *ns* | *ns* |
| **F0-Bi/F2-N, F0-Pat/F2-V+** | **0.017** | **0.034** | *ns* | *ns* | *ns* | *ns* | *ns* | *ns* | *ns* | *ns* |
| **F0-Bi/F2-N, F0-Pat/F2-T+** | *ns* | *ns* | *ns* | *ns* | *ns* | *ns* | *ns* | *ns* | *ns* | *ns* |
| **F0-Bi/F2-N, F0-Pat/F2-N** | *ns* | *ns* | *ns* | *ns* | *ns* | *ns* | *ns* | *ns* | *ns* | *ns* |
| **F0-Bi/F2-N, F0-N/F2-V+** | **0.001** | **0.001** | *ns* | *ns* | *ns* | *ns* | *ns* | *ns* | *ns* | *ns* |
| **F0-Bi/F2-N, F0-N/F2-T+** | **0.01** | **0.013** | *ns* | *ns* | *ns* | **0.054** | *ns* | *ns* | *ns* | *ns* |
| **F0-Bi/F2-N, F0-N/F2-N** | **0.007** | **0.004** | *ns* | *ns* | *ns* | **0.003** | *ns* | *ns* | *ns* | *ns* |
| **F0-Mat/F2-V+, F0-Mat/F2-T+** | *ns* | *ns* | *ns* | *ns* | *ns* | *ns* | *ns* | *ns* | *ns* | *ns* |
| **F0-Mat/F2-V+, F0-Mat/F2-N** | *ns* | *ns* | *ns* | *ns* | *ns* | *ns* | *ns* | *ns* | *ns* | *ns* |
| **F0-Mat/F2-V+, F0-Pat/F2-V+** | **0.036** | **0.030** | *ns* | *ns* | *ns* | **0.004** | *ns* | *ns* | *ns* | *ns* |
| **F0-Mat/F2-V+, F0-Pat/F2-T+** | **0.049** | **0.007** | *ns* | *ns* | *ns* | **0.004** | *ns* | *ns* | *ns* | *ns* |
| **F0-Mat/F2-V+, F0-Pat/F2-N** | **0.033** | **0.049** | *ns* | *ns* | *ns* | *ns* | *ns* | *ns* | *ns* | *ns* |
| **F0-Mat/F2-V+, F0-N/F2-V+** | **0.002** | **0.002** | *ns* | *ns* | *ns* | *ns* | *ns* | *ns* | *ns* | *ns* |
| **F0-Mat/F2-V+, F0-N/F2-T+** | **0.005** | **0.002** | *ns* | *ns* | *ns* | *ns* | *ns* | *ns* | *ns* | *ns* |
| **F0-Mat/F2-V+, F0-N/F2-N** | **0.003** | **0.001** | *ns* | *ns* | *ns* | *ns* | *ns* | *ns* | *ns* | *ns* |
| **F0-Mat/F2-T+, F0-Mat/F2-N** | **0.011** | **0.019** | *ns* | *ns* | *ns* | **0.04** | *ns* | *ns* | *ns* | *ns* |
| **F0-Mat/F2-T+, F0-Pat/F2-V+** | **0.025** | **0.028** | *ns* | *ns* | *ns* | **0.004** | *ns* | *ns* | *ns* | *ns* |
| **F0-Mat/F2-T+, F0-Pat/F2-T+** | **0.033** | **0.04** | *ns* | *ns* | *ns* | **0.010** | *ns* | *ns* | *ns* | *ns* |
| **F0-Mat/F2-T+, F0-Pat/F2-N** | **0.016** | **0.044** | *ns* | *ns* | *ns* | **0.003** | *ns* | *ns* | *ns* | *ns* |
| **F0-Mat/F2-T+, F0-N/F2-V+** | **0.002** | **0.001** | *ns* | *ns* | *ns* | **0.014** | *ns* | *ns* | *ns* | *ns* |
| **F0-Mat/F2-T+, F0-N/F2-T+** | **0.008** | **0.003** | *ns* | *ns* | *ns* | **0.008** | *ns* | *ns* | *ns* | *ns* |
| **F0-Mat/F2-T+, F0-N/F2-N** | **0.009** | **0.003** | *ns* | *ns* | *ns* | **0.001** | *ns* | *ns* | *ns* | *ns* |
| **F0-Mat/F2-N, F0-Pat/F2-V+** | **0.003** | **0.001** | *ns* | *ns* | *ns* | **0.007** | *ns* | *ns* | *ns* | *ns* |
| **F0-Mat/F2-N, F0-Pat/F2-T+** | **0.017** | **0.012** | *ns* | *ns* | *ns* | *ns* | *ns* | *ns* | *ns* | *ns* |
| **F0-Mat/F2-N, F0-Pat/F2-N** | **0.034** | **0.025** | *ns* | *ns* | *ns* | *ns* | *ns* | *ns* | *ns* | *ns* |
| **F0-Mat/F2-N, F0-N/F2-V+** | **0.001** | **0.001** | *ns* | *ns* | *ns* | *ns* | *ns* | *ns* | *ns* | *ns* |
| **F0-Mat/F2-N, F0-N/F2-T+** | **0.001** | **0.002** | *ns* | *ns* | *ns* | *ns* | *ns* | *ns* | *ns* | *ns* |
| **F0-Mat/F2-N, F0-N/F2-N** | **0.001** | **0.001** | *ns* | *ns* | *ns* | *ns* | *ns* | *ns* | *ns* | *ns* |
| **F0-Pat/F2-V+, F0-Pat/F2-T+** | *ns* | *ns* | *ns* | *ns* | *ns* | *ns* | *ns* | *ns* | *ns* | *ns* |
| **F0-Pat/F2-V+, F0-Pat/F2-N** | *ns* | *ns* | *ns* | *ns* | *ns* | *ns* | *ns* | *ns* | *ns* | *ns* |
| **F0-Pat/F2-V+, F0-N/F2-V+** | **0.004** | **0.008** | *ns* | *ns* | *ns* | **0.001** | *ns* | *ns* | *ns* | *ns* |
| **F0-Pat/F2-V+, F0-N/F2-T+** | **0.042** | *ns* | *ns* | *ns* | *ns* | **0.001** | *ns* | *ns* | *ns* | *ns* |
| **F0-Pat/F2-V+, F0-N/F2-N** | **0.003** | **0.003** | *ns* | *ns* | *ns* | **0.001** | *ns* | *ns* | *ns* | *ns* |
| **F0-Pat/F2-T+, F0-Pat/F2-N** | *ns* | *ns* | *ns* | *ns* | *ns* | *ns* | *ns* | *ns* | *ns* | *ns* |
| **F0-Pat/F2-T+, F0-N/F2-V+** | **0.022** | **0.027** | *ns* | *ns* | *ns* | *ns* | *ns* | *ns* | *ns* | *ns* |
| **F0-Pat/F2-T+, F0-N/F2-T+** | **0.033** | **0.023** | *ns* | *ns* | *ns* | **0.054** | *ns* | *ns* | *ns* | *ns* |
| **F0-Pat/F2-T+, F0-N/F2-N** | *ns* | *ns* | *ns* | *ns* | *ns* | **0.057** | *ns* | *ns* | *ns* | *ns* |
| **F0-Pat/F2-N, F0-N/F2-V+** | **0.001** | **0.006** | *ns* | *ns* | *ns* | **0.051** | *ns* | *ns* | *ns* | *ns* |
| **F0-Pat/F2-N, F0-N/F2-T+** | **0.02** | **0.038** | *ns* | *ns* | *ns* | *ns* | *ns* | *ns* | *ns* | *ns* |
| **F0-Pat/F2-N, F0-N/F2-N** | **0.008** | **0.014** | *ns* | *ns* | *ns* | **0.018** | *ns* | *ns* | *ns* | *ns* |
| **F0-N/F2-V+, F0-N/F2-T+** | *ns* | *ns* | *ns* | *ns* | *ns* | *ns* | *ns* | *ns* | *ns* | *ns* |
| **F0-N/F2-V+, F0-N/F2-N** | *ns* | *ns* | *ns* | *ns* | *ns* | *ns* | *ns* | *ns* | *ns* | *ns* |
| **F0-N/F2-T+, F0-N/F2-N** | *ns* | *ns* | *ns* | *ns* | *ns* | *ns* | *ns* | *ns* | *ns* | *ns* |
